# Potential Anti-Tumor Activity of Nardoguaianone L Isolated from *Nardostachys jatamansi* DC. in SW1990 Cells

**DOI:** 10.3390/molecules27217490

**Published:** 2022-11-03

**Authors:** Chun-Yan Sang, Yi-Dan Zheng, Li-Mei Ma, Kai Wang, Cheng-Bo Wang, Tian Chai, Komila A. Eshbakova, Jun-Li Yang

**Affiliations:** 1 CAS Key Laboratory of Chemistry of Northwestern Plant Resources and Key Laboratory for Natural Medicine of Gansu Province, Lanzhou Institute of Chemical Physics, Chinese Academy of Sciences (CAS), Lanzhou 730000, China; 2 College of Life Science, Northwest Normal University, Lanzhou 730070, China; 3 Beijing Research Institute, University of Chinese Academy of Sciences, Beijing 100049, China; 4 S. Yu. Yunusov Institute of the Chemistry of Plant Substances, Academy of Sciences, Tashkent 100170, Uzbekistan

**Keywords:** natural products, *Nardostachys jatamansi* DC., pancreatic cancer, iTRAQ/TMT, MET/PTEN/TGF-β pathway

## Abstract

Natural products (NPs) were a rich source of diverse bioactive molecules. Most anti-tumor agents were built on natural scaffolds. *Nardostachys jatamansi* DC. was an important plant used to process the traditional Chinese herbal medicines “gansong”. Pancreatic cancer was the fourth most common cause of cancer-related death in the world. Hence, there was an urgent need to develop novel agents for the treatment of pancreatic cancer. In this paper, nardoguaianone L (G-6) is isolated from *N. jatamansi*, which inhibited SW1990 cells colony formation and cell migration, and induced cell apoptosis. Furthermore, we analyzed the differential expression proteins after treatment with G-6 in SW1990 cells by using iTRAQ/TMT-based quantitative proteomics technology, and the results showed that G-6 regulated 143 proteins’ differential expression by GO annotation, including biological process, cellular component, and molecular function. Meanwhile, KEGG enrichment found that with Human T-cell leukemia virus, one infection was the most highly enhanced pathway. Furthermore, the MET/PTEN/TGF-β pathway was identified as a significant pathway that had important biological functions, including cell migration and motility by PPI network analysis in SW1990 cells. Taken together, our study found that G-6 is a potential anti-pancreatic cancer agent with regulation of MET/PTEN/TGF-β pathway.

## 1. Introduction

Various traditional Chinese medicines and their associated active compounds have been remarked to exhibit powerful anti-cancer activities and important health benefits, including plant origin compounds such as alkaloids, terpenoids, flavonoids, plant polysaccharides, polyphenols, organic acids and lignans [1,2,3]. Currently, plant drugs including liposomal doxorubicin, paclitaxel, bleomycin, vinblastine, vincristine, and etoposide are used in clinical practice [4]. Between 1981 and 2019, nearly 50% of marketed small-molecular drugs have the molecular structures or core pharmacodynamic structures deriving from natural products [5]. The herb of *Nardostachys jatamansi* DC. could be one important resource for the discovery of bioactive natural products [6], including anti-depressant [7], antioxidant [8], anti-tumor [9], anti-infammatory [10], anti-Parkinson [11], and oxidative stress [12]. However, the bioactive substances and mechanisms of action of *N. jatamansi* have yet to be identified.

We extracted and isolated novel compounds from roots and rhizomes of *N. jatamansi*, tested their inhibition on human pancreatic cancer cell lines growth [13], and found nardoguaianone L (G-6) from *N. jatamansi* as a compound with original structure and a set of interesting biological properties [14]. We detected that G-6 inhibited SW1990 cells colony formation migration and induced cell apoptosis, acting as a potential therapeutic agent. Furthermore, we analyzed the differential expression proteins when SW1990 cells treated with or without by G-6 using iTRAQ/TMT-based quantitative proteomics technology. The received protein data were examined using bioinformatics methods in an effort to extract information relevant to involved pathways. The differential protein expression in biological process (BP), cell component (CC), and molecular function (MF) categories by gene ontology (GO) analysis using theKEGG database were used to annotate the protein pathway. The aim of this paper was to evaluate the anti-tumor effects of G-6 and its potential application in pancreatic cancer chemotherapy.

## 2. Results

### 2.1. G-6 Inhibited SW1990 Cells Proliferation and Colony Formation

**Figure 1:**
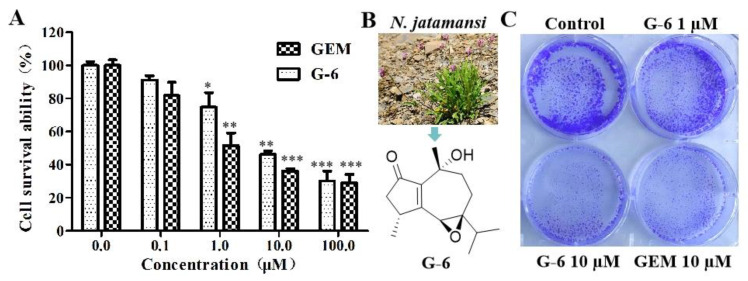
G-6 inhibited SW1990 cells proliferation and colony formation. (**A**) The effect of G-6 inhibiting SW1990 cells proliferation. SW1990 cells were treated with various concentrations of G-6 for 72 h, respectively, with GEM as positive control. Data shown are means ± SEM (*n* = 3). * *p* < 0.05, ** *p* < 0.01, *** *p* < 0.001 vs. 0 µM. (**B**) The structure of G-6 isolated from N. jatamansi. (**C**) The effect of G-6 inhibiting SW1990 cells colony formation. SW1990 cells were treated with 1 and 10 µM of G-6 for 24 h, and stained with crystal violet, with GEM 10 µM as positive control.

**Figure 2:**
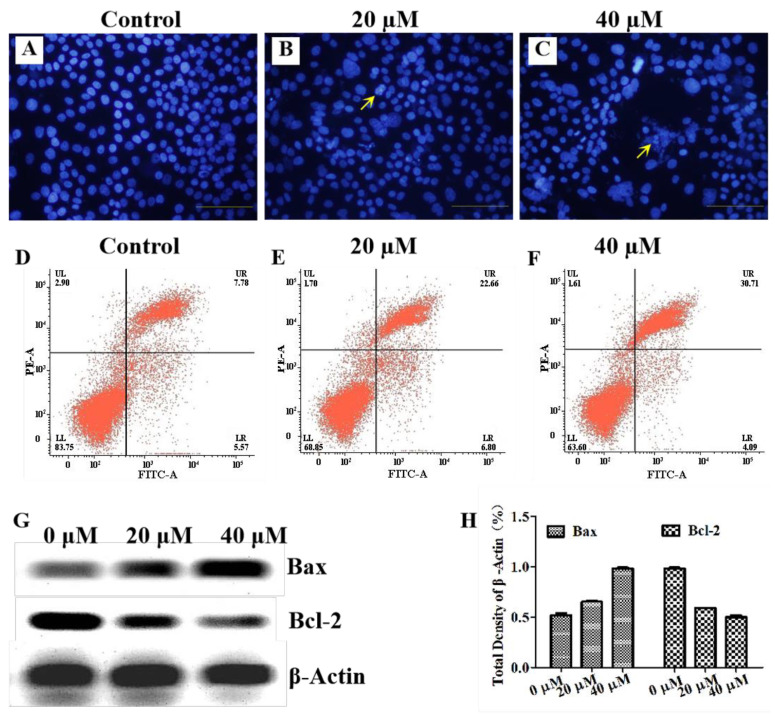
G-6 induced SW1990 cells apoptosis. (**A**–**C**): Representative images of SW1990 cells treated with DMSO or G-6 (20 μM and 40 μM) in cell morphology assay for 24 h. (**D**–**F**): Representative images for SW1990 cells treated with DMSO or G-6 (20 μM and 40 μM) for 24 h prior to staining with Annexin V-FITC/PI staining followed by flow cytometry analysis. (**G**,**H**): Representative Western blotting assay images are shown for SW1990 cells treated with DMSO or G-6 (20 μM and 40 μM) for 24 h and immunoblotting with anti-Bax, and anti-Bcl-2. Data shown are means ± SEM (*n* = 3).

**Figure 3:**
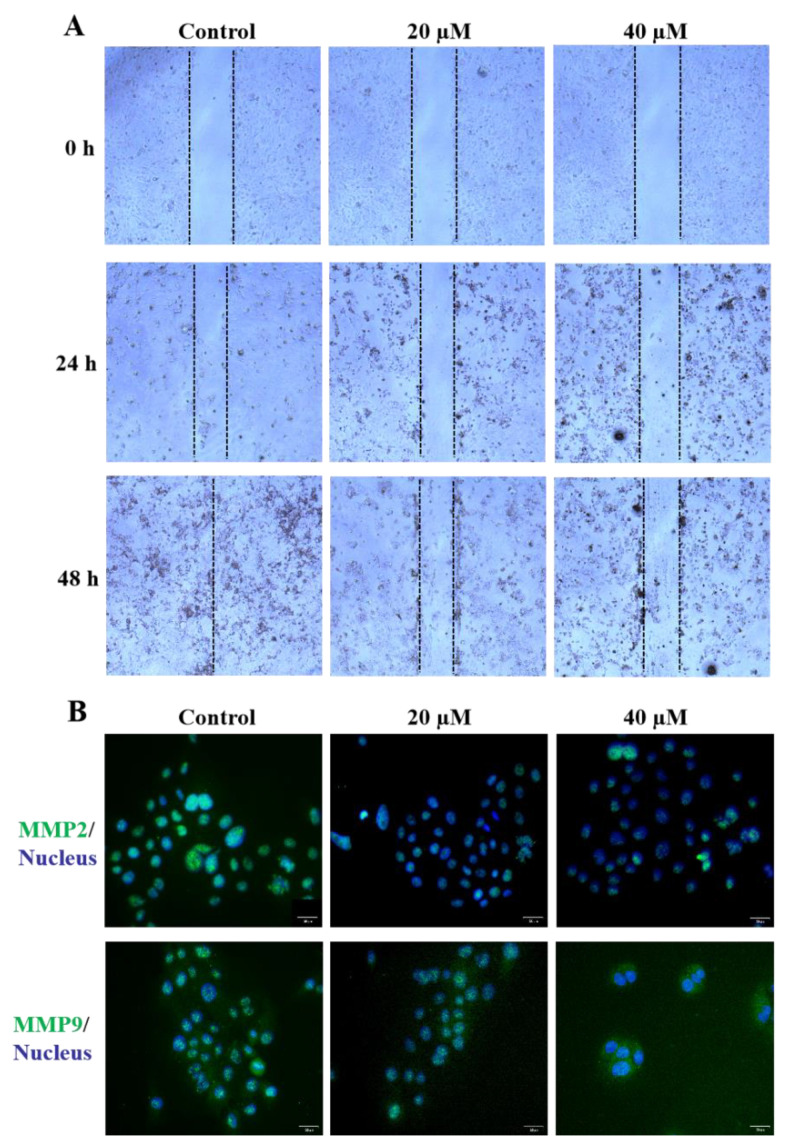
Effects of G-6 on in vitro SW1990 cells migration. (**A**) Effect of G-6 on the wound healing assay of SW1990 cells. Cells were seeded into six-well plates and treated with different concentrations of G-6 for 24 h and 48 h. (**B**) Representation of immunofluorescent images of SW1990 cells treated with DMSO or G-6 at various concentrations for 24 h. SW1990 cells were stained with MMP2 or MMP9 (green), and DNA (blue). Overlapping localization is shown in the merged images.

**Figure 4:**
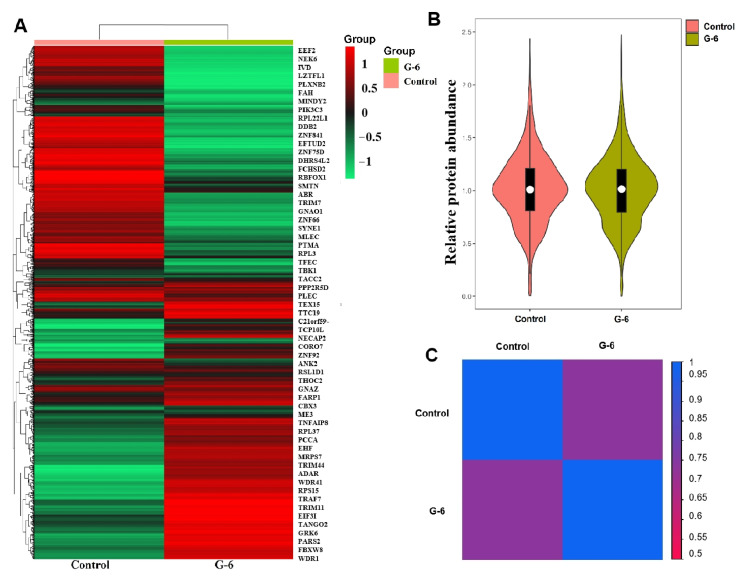
Quantitative analysis of protein of SW1990 cell treated with G-6 10 µM for 24 h or without. (**A**) Heatmap of the expression levels of total proteins in SW1990 cells. The red-colored clusters represent up-regulated proteins, and the green-colored clusters represent down-regulated proteins. (**B**) A violin plot showing the distribution pattern of relative protein abundance was similar and sample pre-treatment was stable. (**C**) Correlation analysis of samples (relativity weakened with color from blue to red).

**Figure 5:**
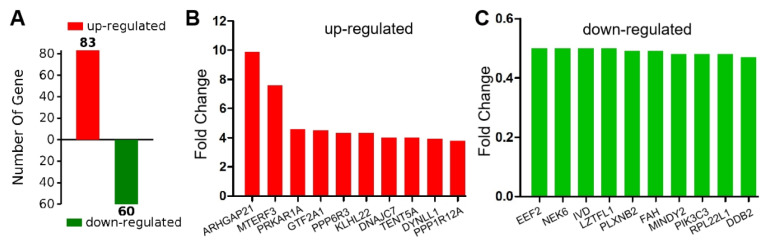
The histogram shows the number of upregulated and downregulated proteins in SW1990 cells by treating G-6 compared with control. (**A**) The number of significant differential abundance was also statistic and displayed by the positive and negative column figure; (**B**) The top 10 of up-regulated proteins; (**C**) The top 10 of down-regulated proteins.

**Figure 6:**
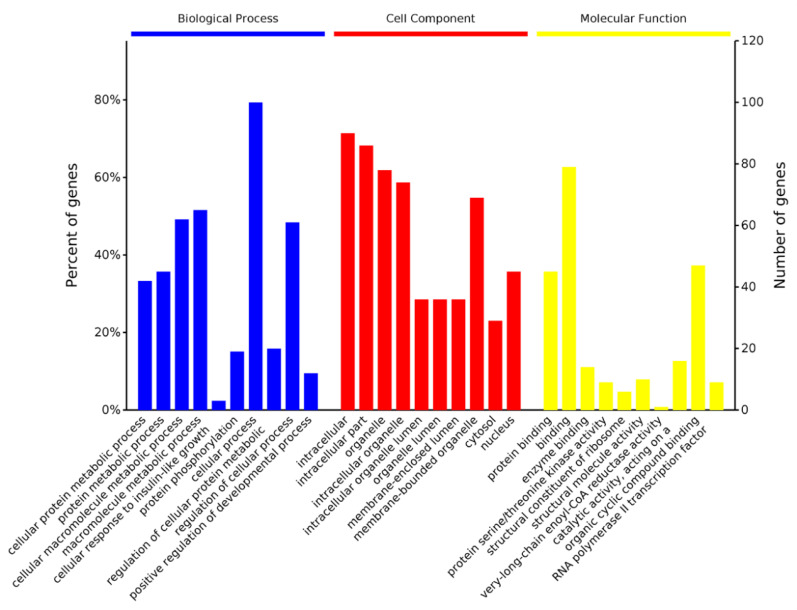
GO is annotation of identified in SW1990 cells treatment with G-6 10 µM for 24 h proteins in three categories: biological process (BP), cellular component (CC) and molecular function (MF).

To evaluate the effect of G-6 (Figure 1B) on tumor cell viability, pancreatic cancer cell lines were treated with G-6 for 72 h, and cell viability was studied using the MTT assay, with gemcitabine (GEM) as positive control. As illustrated in Figure 1A, G-6 showed a remarkable inhibition on pancreatic cancer cell viability, and the IC_50_ value was 2.1 ± 0.3 μM; it’s similar to that of GEM (1.8 ± 0.5 μM). Furthermore, in order to study the G-6’s inhibition performance towards SW1990 cells’ proliferation, SW1990 cells were analyzed using a cell clone formation trial. The constructed cell colonies were numbered after 10 days. Compared with control, G-6 decreased number of cell colonies with 1 μM and 10 μM, and GEM as a positive control group (Figure 1C).

### 2.2. G-6 Induced Cell Apoptosis in SW1990 Cells

Given the unique architecture and promising biological activities, as well as the potential to elicit new biological discoveries, the effects of G-6 inducing cell apoptosis of SW1990 cells were assessed with the cell morphology assay. As shown in Figure 2A–C, the Hoechst 33258 staining confirmed that G-6 induced a regular apoptotic character in cells, including cell detachment and shrinkage, and nuclear fragmentation and condensation. Such findings were confirmed by using flow cytometric Annexin V/PI assay (Figure 2D–F); G-6 induced SW1990 cells apoptosis at both 20 and 40 μM (22.66% and 30.71%, respectively) when compared to the control (13.35%). The above findings revealed that G-6 operated its anti-proliferative effects possibly by inducing apoptosis in SW1990 cells. Furthermore, the underlying molecular mechanisms were then conducted using western blot assay (Figure 2G,H). Mitochondria represent the central checkpoint in propagating apoptotic signaling pathways, and it can regulate apoptosis-related proteins such as Bax and Bcl-2 to enter mitochondria, which induced the mitochondria to release cytochromec, finally triggering the execution of apoptosis. G-6 significantly up-regulated the expression of Bax, together with down-regulation of Bcl-2. Thus, it was illuminated that G-6 induced cell apoptosis via the mitochondria-dependent pathway. 

### 2.3. Effects of G-6 on In Vitro SW1990 Cells Migration

One of the striking features of SW1990 cells is their heightened capacity to invade normal tissue. Matrix metalloproteinases 2 and 9 (MMP2 and MMP9) are involved in extracellular matrix degradation and can both activate latent TGF-β [15]. Therefore, we first executed a scratch-induced migration assay for G-6. From Figure 3A, G-6 inhibited the cells movement into the impaired region and declined the cell number in the wound relative to the vehicle-treated SW1990 cells. SW1990 cells treated with G-6 (20 and 40 μM) for 0 h, 24 h and 48 h in the wounds were non-confluent in a dose or time-dependent accordingly, confirming a striking impairment of SW1990 cells migration by G-6. Furthermore, the densitometric quantification of MMP2 and MMP9 expression was observed by immunofluorescent staining (Figure 3B).

### 2.4. Quantitative Analysis of Protein

Sample correlation diagram analyzed by TMT-based proteomics. Violin-plot study was conducted using the OmicShare tools, a free online platform for data analysis (https://www.omicshare.com/tools/, accessed on 10 July 2022). Using a clustermap generated from data analysis (http://www.bioinformatics.com.cn, accessed on 10 July 2022), we further show a similar pattern by plotting a heatmap for each event (Figure 4A), which indicated the differentially expressed proteins in control and G-6 groups; the distribution pattern of relative protein abundance is very similar, indicating that the pre-treatment and instrument status are very stable (Figure 4B).The correlation analysis of the heatmap was a graph that analyzed the correlation between control and G-6 (Figure 4C).

### 2.5. Analysis for Differentially Expressed Proteins (DEPs)

Gene Ontology (GO) is community-based bioinformatics resource that supplies information about gene product function using ontologies to represent biological knowledge. To understand proteomic change during follicular development and maturation among the above two groups, we screened reliable proteins and differential proteins. A total of 9864 reliable proteins were found in this study. The number of significant differential abundance was also a statistic and displayed by the positive and negative column figure (Figure 5A). For instance, there were 83 upregulated proteins (red) and 60 downregulated proteins (green) in G-6 group. The top 10 of up-regulated proteins including ARHGAP21 (Rho GTPase-activating protein 21), MTERF3 (Transcription termination factor 3, mitochondrial), PRKAR1A (cAMP-dependent protein kinase type I-alpha regulatory subunit), GTF2A1 (Transcription initiation factor IIA subunit 1), PPP6R3 (Serine/threonine-protein phosphatase 6 regulatory subunit 3), KLHL22 (Kelch-like protein 22), DNAJC7 (DnaJ homolog subfamily C member 7), TENT5A (Terminal nucleotidyltransferase 5A), DYNLL1 (Dynein light chain), PPP1R12A (Protein phosphatase 1 regulatory subunit 12A) (Figure 5B). The top 10 of down-regulated proteins including EEF2 (Elongation factor 2), NEK6 (Serine/threonine-protein kinase Nek6), IVD (Isovaleryl-CoA dehydrogenase, mitochondrial), LZTFL1 (Leucine zipper transcription factor-like protein 1), PLXNB2 (Plexin-B2), FAH (Fumarylacetoacetase), MINDY2 (Ubiquitin carboxyl-terminal hydrolase), PIK3C3 (Phosphatidylinositol 3-kinase catalytic subunit type 3), RPL22L1 (60S ribosomal protein L22-like 1), DDB2 (DNA damage-binding protein 2) (Figure 5C).

The differentially expressed proteins in molecular function (MF), cell component (CC), and biological process (BP) and categories by GO study, accordingly, was illustrated in Figure 6. In the BP study, a major part of announced proteins was categorized into cellular process, macromolecule metabolic process, cellular macromolecule metabolic process and regulation of cellular process. The CC study revealed that a large number of expressed proteins were attributed to intracellular, intracellular part and organelle. Molecular functional categorization of such proteins exhibited that most of them were found in organic cyclic compound binding and protein binding. The data analysis of the GO result confirmed that these G-6-treated associated cellular proteins illustrated an extensive kind of cellular distributions and functions, in accordance with the fact that G-6 participated in multiple indispensable activities via regulation the components of cells.

**Figure 7:**
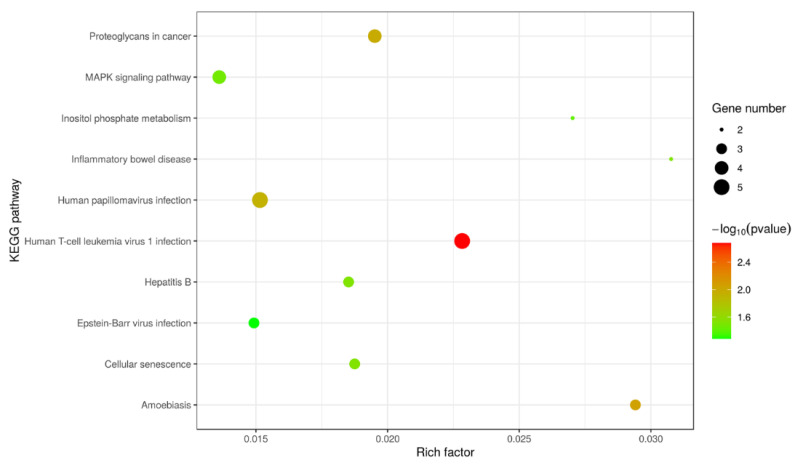
Bubble map of the enriched KEGG pathways of DEP in SW1990 cells treatment with G-6 10 µM for 24 h. The top 10 result pathways with KEGG enrichment were shown in the bubble map and bubble diagram of differential proteins enriched in various pathways. Bubble diameters represent the number of proteins in each pathway. The color represents the magnitude of significance of enrichment.

**Figure 8:**
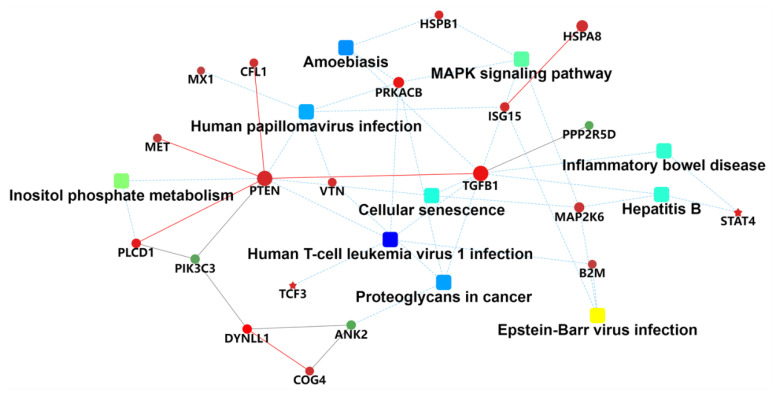
Analysis and enrichment results of KEGG pathways and protein-protein interaction (PPI) in SW1990 cells treatment with G-6 10 µM for 24 h. The PPI analysis was based on fold change of protein, protein-protein interaction, KEGG pathway enrichment and biological process enrichment. Circle nodes refer to proteins. Rectangle refers to KEGG pathway or biological process, which were colored with gradient color from green to red (low-high fold change).

The KEGG pathway study was applied to associate protein functions to metabolic process. The KEGG study showed that the highly active pathways that participated were in accordance with G-6 treated with or without in SW1990 cells. As shown in Figure 7, KEGG enrichment was assessed by Rich Factor, *p*-value and the number of proteins enriched to this pathway, with the 10 pathways showing the most significant enrichment. Among them, Human T-cell leukemia virus 1 infection was the most significantly enriched pathway. The PPI network analysis found that some DEPs interact with each other, such as MET/PTEN/TGF-*β*. These key focus hubs expressed a crucial biological activities biological adhesion, cell migration, enzyme activity and biological regulation (Figure 8).

## 3. Discussion

Medicinal plants have been used as medicines or food supplements for the treatment of a wide variety of diseases in China and other Asian countries for quite a long time [16]. Natural products and their structures have a long tradition as valuable source for medicinal chemistry and drug discovery [17]. In consideration of very diversified chemical structures and biological activities of plant secondary metabolites, medicinal plants have become a promising medicinal resource with their natural active components [18]. nardoguaianone L (G-6), from *N. jatamansi* DC., can inhibited SW1990 cells proliferation, colony formation, and tumor cell migration. Meanwhile, G-6 significantly upregulated MMP2 and MMP9 expression, which was associated with cell migration. It is well known that tumor growth is composed of a balance between cell proliferation and cell apoptosis [19]. These activities are regulated by many factors such as apoptosis proteins [20], including Bax and Bcl-2 [21]. However, Bcl-2 also bind to Bax proteins and affect Bax function [22]. Bax knockdown significantly suppressed induced apoptosis [23]. To identify whether G-6 inhibited SW1990 cells proliferation caused by an increased apoptosis and regulation of apoptosis proteins, we subsequently performed Annexin V/PI apoptotic assay and clone formation assay. It was found that G-6 significantly induced cell apoptosis. Consistently, G-6 not only upregulated Bax expression but also downregulated Bcl-2 expression.

In addition, iTRAQ/TMT-based quantitative proteomics [24] was used to analyze and compare proteomic changes of SW1990 cells treated with G-6, and protein function annotation and differentially expressed protein statistical analysis were performed. The results revealed 143 differentially expressed proteins in SW1990 cells treated with G-6, of which 83 were up-regulated and 60 were down-regulated. These differentially expressed proteins were analyzed by GO annotation, and they were enriched in three aspects: biological process, cellular component, and molecular function. KEGG enrichment found Human T-cell leukemia virus 1 infection was the most significantly enriched pathway. The PPI network analysis found MET/PTEN/TGF-*β* pathway was most significant pathway and have important biological functions including cell migration and motility.

## 4. Materials and Methods

### 4.1. Materials

Nardoguaianone L (G-6) was obtained by our research group, and the HPLC purity >98% [13]. For in vitro assays, G-6 dissolved in DMSO and diluted in the relevant culture media to a final DMSO concentration of 0.1% (*v*/*v*^−1^). Other reagents materials including Dulbecco’s Modified Eagle Medium (DMEM, Gibco, Carlsbad, CA, USA), MTT (3-(4,5-dimethyl-2-thiazolyl)-2,5-diphenyl-2-*H*-tetrazolium bromide, Kum-amoto, Japan), DMSO (Dimethyl sulfoxide) (Sigma, St Louis, MO, USA). The primary antibodies MMP2/9 (Matrix metallopeptidase 2/9) (Cell Signaling Technology, Beverly, MA, USA) and FITC-conjugated Goat Anti-Rabbit IgG (H + L) Secondary Antibody (Proteintech, Chicago, IL, USA).

### 4.2. Cell Culture

SW1990 cells (Chinese Academy of Sciences, Shanghai, China) were cultured in DMEM that contained 100 μg/mL streptomycin, 100 U/mL penicillin (Gibco, Carlsbad, CA, USA) and 10% (*v*/*v*) fetal bovine serum (Sijiqing, Hangzhou, China) in a humidified atmosphere contained 5% CO_2_ at 37 °C.

### 4.3. Cell Viability Assay

Cells were incubated at 37 °C in a 5% CO_2_ atmosphere. Cell viability assessed by MTT assay [25]. G-6 and reference GEM were dissolved in DMSO to prepare five concentrations (0.1–100 μM). The SW1990 cells were plated in 96-well plates and allowed to attach for 4–6 h, then exposed to aquadplex well for 72 h. The MTT solution was added to the cells and the plate was incubated for 4 h and added 100 µL DMSO. The optical density (OD) was then measured at 496 nm using Microplate Reader (Rayto, Shenzhen, China). The cell viability (%) = (OD_570_ of treated group − OD_570_ of blank group)/(OD_570_ of untreated group − OD_570_ of blank group) × 100%.

### 4.4. Immunofluorescence Analysis

SW1990 cells plated on coverslips were either treated or untreated using different levels of compound G-6. After 24 h, the cells were permeabilized in 2% bovine serum albumin and 0.1% Triton X-100 and fixed with 4% paraformaldehyde as illustrated in previous work [26]. The samples were incubated over night with anti-MMP2/9 antibody (Cell Signaling Technology, Beverly, MA, USA) at 4 °C, then incubated with FITC-conjugated secondary antibody (Proteintech, Chicago, IL, USA) for 2 h at room temperature. The nuclear DNA was then stained with DAPI (Solarbio, Beijing, China). Cells were analyzed under a confocal microscope (Olympus, Tokyo, Japan).

### 4.5. Morphological Analysis

For the morphological investigation, SW1990 cells (1 × 10^5^) were grown on glass slides to 70–80% confluency, and were then treated with 20 µM and 40 µM of compound G-6 for 24 h at 37 °C in an atmosphere containing 5% CO_2_. Following treatment, the cells were fixed, washed twice with phosphate-buffered saline (PBS) and stained with Hoechst 33258 (Solarbio, Beijing, China) staining solution, according to the manufacturer’s protocol [27]. Morphological changes and chromosomal condensation were noticed and quantized using an Olympus BX61 fluorescence microscope (Olympus, Tokyo, Japan).

### 4.6. Apoptosis Assay

SW1990 cells (2 × 10^5^ cells/mL) were plated in 6-well plates and then treated with vehical, 20 μM and 40 μM of compound G-6 for 24 h. The cells apoptosis assay according to protocol of apoptosis kit (Bection Dickinson, San Jose, CA, USA) [28].

### 4.7. Scratch-Induced Migration Assay

SW1990 cells were synchronized in a low serum medium for 8 h, and the cell monolayers were then damaged and created a linear 2 mm width wound. Different concentrations of G-6 were added and the cells were incubated for 0 h, 10 h, 24 h and 48 h. Images were received using a microscope (Olympus, Tokyo, Japan) and the data are declared as the percentage inhibition of migration rate compared with untreated cells [29].

### 4.8. Protein Extraction and Digest

Lysis solution (Thermo Fisher Scientific, Waltham, MA, USA) and Extraction Buffer (Thermo Fisher Scientific, Waltham, MA, USA) were added to each sample [30], and the tissue was ground at 4 °C by ultrasonication on ice for 40 min. and extracted the proteins according to the manufacturer’s instructions. Finally, centrifuge the sample at 10,000× *g* for 10 min, the supernatants were collected, and protein concentrations were determined by Pierce™ BCA Protein Assay Kit. And more steps are referred to Burke et al. [31].

### 4.9. Peptides Labeling and MS Analysis

Ten-plex TMT labeling was performed according to the manufacturer’s instructions (Thermo Fisher Scientific Inc., Waltham, MA, USA). Equilibrate the TMT Label Reagents to room temperature and added 20 μL of anhydrous acetonitrile into each tube with intermittent shaking for 5 min, and centrifuge the tube to gather the solution. Following, TMT Reagent and alkylated protein was mixed and incubated the reaction for 1 h at room temperature. The reaction was quenched when added 1 μL of 5% hydroxylamine to the sample and incubated for 15 min. Finally, Vortex each tube to mix, combine samples at equal amounts in new microcentrifuge tube and stored at −80 °C.

### 4.10. RPLC Analysis

Prepare solutions in 1.5 mL tubes according to requirement. Remove the protective white tip from the bottom of the column and discard. Place the column into a 2.0 mL sample tube. Centrifuge at 5000× *g* for 2 min to remove the solution and pack the resin material. Discard the liquid. Remove the top screw cap and load 300 μL of ACN into the column. Replace the cap, place the spin column back into a 2.0 mL sample tube and centrifuge at 5000× *g* for 2 min. Discard ACN and repeat wash step. Wash the spin column twice with 0.1% TFA solution. The column is now conditioned and ready for use. Dissolve the digested sample in 300 μL of 0.1% TFA solution. Place the spin column into a new 2.0 mL sample tube. Load 300 μL of the sample solution onto the column, replace the top cap and centrifuge at 3000× *g* for 2 min. Retain eluate as “flow-through” fraction. Place the column into a new 2.0 mL sample tube. Load 300 μL of 5% ACN, 0.1% TEA to remove unreacted TMT reagent. Place the column into a new 2.0 mL sample tube. Load 300 μL of the appropriate elution solution and centrifuge at 3000× *g* for 2 min to collect the fraction. Repeat for the remaining step gradient fractions using the appropriate elution solutions in new 2.0 mL sample tubes. Evaporate the liquid contents of each sample tube to dryness using vacuum centrifugation.

### 4.11. UPLC-MS/MS Analysis

Samples were resuspended with Nano-UPLC buffer A (0.1% FA) and buffer B 20% 0.1% formic acid in water-80% acetonitrile. The online Nano-UPLC was employed on the Easy-nLC 1200 System (Thermo Scientific, Waltham, MA, USA). The samples were washed by Nano-UPLC mobile phase at 600 nL/min. An elution gradient of 4–10% acetonitrile (0.1% formic acid) in 5 min, from 10% to 22% B for 80 min, from 22% to 40% B for 25 min, from 40% to 95% B for 5 min and from 95% to 95% B for 5 min. The gradient was used on an analytical column (PepMap100, C18 1.9 μm 150 μm X150 mm manufacture, Beijing, China). Data attainment was carried out using a Orbitrap Eclipse (Thermo Scientific, Waltham, MA, USA) fitted with a Nanospray. The Orbitrap Eclipse instrument was performed with a data-dependent top-20 experiment with 60K resolution for the full MS scans, 30 K resolution for high energy collisional dissociation (HCD) MS/MS scans. A top 20 method was used. Full MS scans were received in the Orbitrap mass analyzer over the range *m*/*z* 350–1500 with a mass resolution of 60,000 (at *m*/*z* 200).

### 4.12. Protein Identification and Quantification

The collected spectral result files were studied via Proteome Discoverer™ 2.5 software by the SEQUEST^®^ search engine, constrained with a precursor mass tolerance of 20 ppm and fragment mass tolerance of 20 ppm [32]. Carbamidomethylation (+57.021 Da) of cysteine and TMT isobaric labeling (+229.163 Da) of lysine were set as static modifications while TMT labeling of the peptide and protein N-termini, methionine oxidation (+15.996 Da). Data was searched against a Uniprot complete Homo sapiens database with 1% false discovery rate (FDR) criteria. For protein quantitation, we required at least two unique spectra. P-SDA was defined by the criteria of a fold change (FC) ≥ 2 or ≤ 0.5 (*p* < 0.05).

### 4.13. Bioinformatics

Gene ontology (GO) and KEGG pathway analysis of the ensemble of detected proteins was performed through OmicsBean (http://www.omicsbean.cn, accessed on 10 July 2022) on online databases.

### 4.14. Data Statistics

All data are repeated at least three parallel experiments. Statistical analyses were performed using GraphPad Prism 8. Results are presented as means ± standard deviation (SD) and compared by Dunn’s Multiple Comparisons Test. *p* < 0.05 was considered statistically significant.

## 5. Conclusions

In conclusion, G-6 from *N. jatamansi* involves multiple mechanisms that culminate in an overall inhibition of tumor growth through both inhibition of proliferation and induction of apoptosis, also including inhibition of tumor cell migration. These results may be explained by the fact that G-6 regulated key factor in quantitative proteomics levels. Therefore, G-6 inhibited the MET/PTEN/TGF-β pathway, mediating these currents contributions to the anti-tumor activity, and showed that G-6 is a promising anti-pancreatic cancer agent candidate potently in vitro. G-6 may act as a new structural framework for the subsequent development of anti-pancreatic cancer agents.

## Data Availability

The datasets used and/or analyzed during the current study are available from the corresponding author on reasonable request.

## Sample Availability

Samples of the compounds are available from the authors.
